# Early Detection of Neural Fatigue in Clinically Healthy Postpartum Mothers Using Functional Near-Infrared Spectroscopy

**DOI:** 10.7759/cureus.97587

**Published:** 2025-11-23

**Authors:** Ryohei Kimura, Tomoyuki Ueda, Tomoko Ohashi, Hiroshi Ogata

**Affiliations:** 1 Human Anatomy and Physiology, Japanese Red Cross Kyushu International College of Nursing, Fukuoka, JPN; 2 Nursing and Social Welfare, Kyushu University of Nursing and Social Welfare, Tamana, JPN; 3 Medical Technology, Teikyo University Fukuoka, Omuta, JPN

**Keywords:** brain activation, cognitive load, edinburgh postnatal depression scale, functional near infrared spectroscopy, maternal mental health, neural fatigue, postpartum depression, prefrontal cortex

## Abstract

Background

Postpartum mothers often experience depressive symptoms and emotional fluctuations that can affect parenting behavior and increase the risk of child maltreatment. Previous studies have reported reduced prefrontal activation in postpartum women; however, few have clarified the temporal changes in neural activity associated with sustained cognitive load. This study aimed to detect early signs of neural fatigue in clinically healthy mothers by measuring prefrontal activation during task performance using near-infrared spectroscopy (NIRS).

Methods

Eight mothers at one month postpartum (mean age: 31.5 years) participated in this study. Using a wearable optical topography device (WOT-220, NeU Corporation, Japan), changes in oxygenated hemoglobin (Δoxy-Hb) in the prefrontal cortex were measured during three verbal fluency tasks (initial syllables “to,” “se,” and “o”) and an arithmetic task (serial subtraction of seven from 100). The Wilcoxon signed-rank test was used to compare task and calibration periods, and linear regression analysis was conducted to evaluate temporal changes.

Results

All participants exhibited task-related changes in Δoxy-Hb. In the left prefrontal cortex, activation was observed during the initial tasks; however, three out of eight mothers showed a gradual decrease in oxygenation responses as the tasks were repeated and sustained. Among these three, one mother had a relatively high Edinburgh Postnatal Depression Scale (EPDS) score, indicating a depressive tendency, whereas the other two were classified as non-depressed by self-report. Task performance (number of words produced and subtraction counts) was largely maintained, suggesting the presence of “latent fatigue,” in which neural activity declines despite preserved behavioral performance.

Conclusions

This study demonstrated that even clinically healthy postpartum mothers may exhibit reduced sustainability of prefrontal activation under continuous cognitive load. The findings visualize early signs of neural fatigue that are undetectable through self-reported scales. Although this study does not propose NIRS as a diagnostic screening tool, it highlights that some mothers deemed clinically healthy may possess latent vulnerability to fatigue. Early recognition and preventive support based on such hidden fatigue could help prevent the onset of depression, improve the quality of life (QOL) for both mothers and infants, and contribute to the primary prevention of child abuse.

## Introduction

After childbirth, women undergo physical recovery while simultaneously being exposed to multiple psychosocial stressors such as hormonal fluctuations, sleep deprivation, and childcare-related stress. Under these circumstances, postpartum depression (PPD) has been reported to occur in approximately 10-20% of mothers [1], and it may adversely affect mother-infant bonding and infant development [2]. Recently, attention has been drawn to the fact that even mothers who appear clinically healthy and show no overt depressive symptoms may experience latent neural fatigue or reduced emotional regulation capacity [3].

Such subtle alterations in brain function are often difficult to detect using conventional self-reported questionnaires or observational assessments, emphasizing the need for objective physiological indicators for early identification [4]. Among these, functional near-infrared spectroscopy (fNIRS) is a non-invasive method that allows real-time evaluation of prefrontal cortex activity and has been widely applied in neurophysiological studies on emotion, motivation, and fatigue [5]. The prefrontal cortex (PFC) is particularly involved in attention, executive functions, and emotional regulation, and previous studies have shown that oxyhemoglobin (oxy-Hb) responses tend to decrease under conditions of mental fatigue or stress [6].

Furthermore, difficulties in maternal emotional regulation and stress coping have been identified as risk factors for parenting difficulties and neglect, which are closely related to child abuse [7]. Since child maltreatment prevention should focus not only on post-incident interventions but also on identifying risk factors at an early stage [8], visualizing early indicators of “neural fatigue” or diminished emotional regulation in clinically healthy postpartum mothers may serve as a useful approach to capture precursors of elevated abuse risk.

In this study, we assessed prefrontal cortex activity during task performance using fNIRS in postpartum mothers who were clinically evaluated as healthy. By visualizing latent fatigue states that cannot be fully captured by subjective depression scales, this study aimed to provide insights for developing new models of postpartum mental health support and early prevention of child maltreatment.

## Materials and methods

Participants

This study was conducted in the obstetric ward of a general hospital in Fukuoka Prefecture, Japan. Participants were eight mothers who had delivered between March 2023 and March 2024 and attended their 1-month postpartum checkup. All participants received an explanation of the study’s purpose and procedures and provided written informed consent. The study was approved by the Institutional Review Board of the Japanese Red Cross Kyushu International College of Nursing (approval No. 21-018).

The mean age of participants was 31.5 years (range: 24-38), and all were multiparous. Because postpartum depression can occur in both primiparous and multiparous women [9] and previous longitudinal research has shown that depressive symptoms during pregnancy often persist into the postpartum period [10], we analyzed clinically healthy mothers, including multiparas.

Maternal psychological status was assessed using the Japanese version of the Edinburgh Postnatal Depression Scale (EPDS). The EPDS comprises 10 items (total score 0-30), with a cutoff of ≥ 9 indicating possible depressive tendency. The Japanese version can be used without formal permission. For participants with EPDS scores ≥ 9, participation eligibility was determined after discussion among the investigators and the attending physician.

Exclusion criteria included mothers currently receiving treatment for psychiatric disorders and individuals requiring a surrogate decision-maker, based on considerations of ongoing treatment and ethical safeguards. If any participant reported discomfort (e.g., malaise) during measurement, the session was immediately interrupted; if symptoms did not improve, the assessment was terminated, and medical consultation was considered as needed. These determinations were made comprehensively based on the participant’s wishes, investigator observations, and, when necessary, advice from a collaborating physician.

Device and tasks

Prefrontal brain activity was measured using a wearable optical topography system (WOT-220; NeU Corporation, Japan). The device employs two wavelengths (690 and 830 nm) to detect oxygenated hemoglobin (oxy-Hb) and deoxygenated hemoglobin (deoxy-Hb). Probes were positioned over the left prefrontal cortex according to the international 10-20 system. Prior research examining the relationship between EPDS scores and NIRS-derived cerebral blood flow has shown smaller hemodynamic changes in mothers with higher depressive scores and increased oxy-Hb in those with lower scores [11]. The validity of the NIRS device used in this study has been demonstrated in previous development studies [12], and the optimization of wavelength combinations and signal-to-noise ratios has also been reported [13].

Optical intensity signals were sampled at 10 Hz and converted to concentration changes using the modified Beer-Lambert law with standard differential pathlength factors. Changes in oxy-Hb concentration (Δoxy-Hb) were expressed in arbitrary units (au). To reduce slow drifts and physiological noise, data were band-pass filtered (0.01-0.2 Hz) and visually screened for motion artifacts.

**Figure 1:**
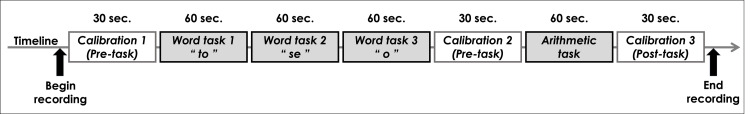
NIRS Measurement Protocol and Task Design Schematic overview of the NIRS task sequence. Participants completed three Word Tasks (WT1–WT3) using the initial syllables “to,” “se,” and “o,” followed by one Arithmetic Task (AT) involving serial subtraction of seven from 100. Calibration (rest) periods were inserted before, between, and after the tasks. Changes in oxygenated hemoglobin (Δoxy-Hb) were continuously recorded throughout the session.

While seated, participants completed three verbal fluency tasks (Word Tasks; WT1-WT3) and one Arithmetic Task (AT). The total measurement time was approximately 10 minutes. In the verbal fluency tasks, participants were instructed to generate as many Japanese words as possible beginning with the initial syllables “to,” “se,” and “o,” selected based on high frequency in daily Japanese and ease of articulation [14]. For the arithmetic task, participants performed serial subtraction of seven from 100 for 1 minute. This task is known to reliably activate the prefrontal cortex as a mental workload paradigm [15] and is widely used in NIRS research. The experimental protocol is illustrated in Figure 1.

A 20-second resting period was provided before and after each task, and changes in oxy-Hb concentration (Δoxy-Hb) during task periods and the corresponding baseline periods were measured. Data were processed using the wearable optical topography (WOT) analysis software, and mean changes relative to the baseline period were calculated. Optical intensity signals were converted using the modified Beer-Lambert law, and Δoxy-Hb was expressed in arbitrary units (au) based on the device output.

Statistical analysis

The primary outcome was the change in oxy-Hb (Δoxy-Hb). Mean values during calibration (baseline) and each task period were compared using the Wilcoxon signed-rank test. Temporal changes within each task were evaluated using linear regression analysis. Statistical analyses were performed with IBM SPSS Statistics version 29 (IBM Corp., Armonk, NY, USA), with a two-tailed significance level of p < 0.05. Python 3.11 was used for visualization and plotting of regression lines. The analysis code used for the linear regression is included in the Appendix.

Ethical considerations

This study was approved by the Institutional Review Board of the Japanese Red Cross Kyushu International College of Nursing (approval No. 21-018). All participants received a full explanation of the study aims and procedures, data protection, voluntary participation, and the right to withdraw, and they provided written informed consent.

## Results

The mean age of the eight participants was 31.5 years (range: 24-38), and all were multiparous. The Edinburgh Postnatal Depression Scale (EPDS) scores ranged from 0 to 7, all of which were below the cutoff score of 9 (Table 1).

**Table 1:** Participant demographics and task results (n = 8). The table presents age, birth order (number of child born in the current delivery; e.g., 1st = first child, 2nd = second child), Edinburgh Postnatal Depression Scale (EPDS) scores, number of words generated in the verbal fluency tasks (WT1–WT3), and number of subtractions performed in the arithmetic task (AT). WT1–WT3 correspond to verbal tasks using the initial syllables “to,” “se,” and “o,” respectively. AT represents the serial subtraction task (100–7). EPDS ≥ 9 indicates a possible depressive tendency.

ID	Age	Birth order	Word task1	Word task2	Word task3	Arithmetic task	EPDS Scores
A	28	3rd	7	5	5	14	0
B	33	4th	7	5	7	14	0
C	24	2nd	6	5	8	3	7
D	28	1st	5	4	5	7	0
E	38	3rd	4	2	4	10	2
F	36	2nd	20	5	9	6	6
G	37	2nd	6	1	4	14	0
H	28	2nd	10	10	10	9	1

Brain activity and task-related changes

Task Performance

Each verbal fluency task (WT1-WT3) required participants to generate as many Japanese words as possible within one minute, beginning with the designated initial syllables “to,” “se,” or “o.” The values shown in Table 1 represent the number of words produced. The arithmetic task (AT) involved serial subtraction of seven from 100 for one minute, and the corresponding values indicate the number of subtractions performed regardless of accuracy.

Changes in Brain Activity During Verbal Tasks (WT1-WT3)

Changes in oxygenated hemoglobin concentration (Δoxy-Hb) were observed in all participants during the verbal tasks (Tables 1 and 2). According to the Wilcoxon signed-rank test, most participants showed significant increases in oxy-Hb during WT1 and WT2 compared with the calibration period (p < 0.05), indicating prefrontal activation during the initial tasks. However, in WT3, several participants (C, D, F, and H) showed downward changes in oxy-Hb, indicating a tendency toward reduced activity with repeated task performance. Participant E showed negative Δoxy-Hb values across all tasks, suggesting decreased ability to maintain activation and reduced tolerance to mental workload. These findings indicate that while prefrontal activity is initially activated, it tends to decline with repeated tasks. This pattern may reflect early signs of neural fatigue in postpartum mothers who are otherwise clinically healthy.

**Table 2:** Results of the Wilcoxon Signed-Rank Test Results of the Wilcoxon signed-rank test comparing changes in oxygenated hemoglobin (Δoxy-Hb) in the prefrontal cortex (average of channels 13–22) between the calibration (resting) period and each task condition. The table presents the median, standard deviation, Z value, and significance level (p-value) for each task condition (WT1, WT2, WT3, AT). The significance level was set at p < 0.05 (two-tailed).WT1–WT3 represent the three verbal fluency tasks, and AT represents the arithmetic task (serial subtraction of seven from 100).Note: Δoxy-Hb = change in oxygenated hemoglobin concentration; WT = Word Task; AT = Arithmetic Task.

ID	Comparison	Z value	p value	Mean rank of negative ranks	Mean rank of positive ranks
A	WT1 vs. Cal1	-3.134	0.002	52.6	67.27
	WT2 vs. Cal1	-5.239	< 0.001	65.93	50.03
	WT3 vs. Cal1	-2.73	0.006	60.25	63.88
	AT vs. Cal2	-2.715	0.007	82.21	83.49
B	WT1 vs. Cal1	-7.379	< 0.001	50.67	78.25
	WT2 vs. Cal1	-10.4	< 0.001	1.5	73.5
	WT3 vs. Cal1	-10.41	< 0.001	67.17	186.1
	AT vs. Cal2	-10.689	< 0.001	76.99	3
C	WT1 vs. Cal1	-2.645	0.008	63.42	87.37
	WT2 vs. Cal1	-0.75	0.453	74.96	78.35
	WT3 vs. Cal1	-1.003	0.316	75.7	77.49
	AT vs. Cal2	-4.89	< 0.001	65.43	82.45
D	WT1 vs. Cal1	-4.954	< 0.001	58.89	85.93
	WT2 vs. Cal1	-8.861	< 0.001	43.35	82.41
	WT3 vs. Cal1	-4.843	< 0.001	57.84	87.08
	AT vs. Cal2	-10.638	< 0.001	5.17	79.43
E	WT1 vs. Cal1	-4.961	< 0.001	58.96	36.56
	WT2 vs. Cal1	-2.112	0.035	50.9	52.7
	WT3 vs. Cal1	-7.633	< 0.001	19.89	58.8
	AT vs. Cal2	-8.162	< 0.001	99.31	49.79
F	WT1 vs. Cal1	-2.066	0.039	82.58	68.99
	WT2 vs. Cal1	-6.525	< 0.001	94.56	42.77
	WT3 vs. Cal1	-1.025	0.306	93.88	66.36
	AT vs. Cal2	-4.089	< 0.001	62.54	81.37
G	WT1 vs. Cal1	-7.43	< 0.001	49.04	71.53
	WT2 vs. Cal1	-6.743	< 0.001	40.16	77.93
	WT3 vs. Cal1	-5.955	< 0.001	38.35	83.77
	AT vs. Cal2	-1.801	0.072	75.9	83.22
H	WT1 vs. Cal1	-7.452	< 0.001	51.72	81.37
	WT2 vs. Cal1	-6.878	< 0.001	48.95	84.56
	WT3 vs. Cal1	-4.163	< 0.001	63.98	81.08
	AT vs. Cal2	-2.163	0.031	18.08	20.96

Changes in Brain Activity During the Arithmetic Task (AT)

During the arithmetic task, six out of eight participants showed significantly increased oxy-Hb levels compared with the calibration period (p < 0.01). Marked activation was observed in participants A, D, F, G, and H. In contrast, participants B and E exhibited larger negative ranks than positive ranks, indicating a downward trend in oxy-Hb. These results suggest that following transient activation during the verbal tasks, prefrontal activity decreased during the subsequent arithmetic task. In other words, sustained task load led to a decline in neural activation over time, indicating difficulty maintaining activation during prolonged cognitive effort (Table 2).

Evaluation of Temporal Changes by Linear Regression

**Figure 2:**
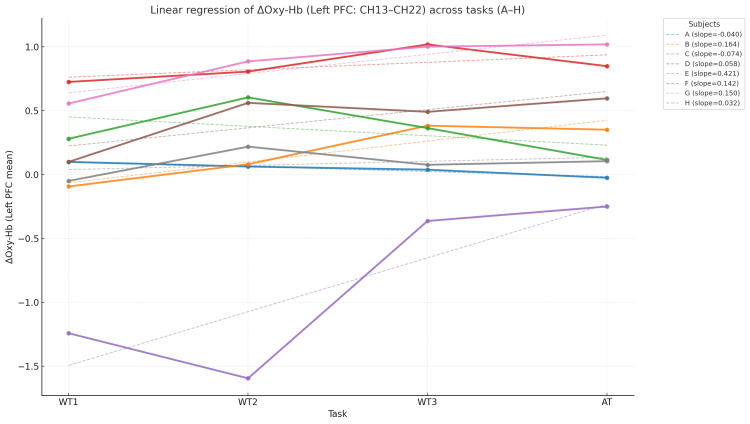
Temporal Changes in Δoxy-Hb in the Left Prefrontal Cortex (Individual Linear Regression for Eight Participants) Mean Δoxy-Hb (arbitrary units, au) across WT1–WT3 and AT with participant-specific regression lines. Solid lines indicate positive slopes; dashed lines indicate negative slopes. A–H: participant IDs; WT1–WT3: verbal fluency tasks using the initial syllables “to,” “se,” and “o”; AT: serial subtraction of seven from 100.

As shown in Figure 2, the mean Δoxy-Hb in the left prefrontal cortex (channels 13-22) was analyzed using linear regression. During the verbal fluency tasks (WT1-WT3), five of eight participants (B, D, E, F, and G) exhibited positive slopes, showing an increasing trend in prefrontal activity with task repetition, while three participants (A, C, and H) showed negative slopes, indicating a gradual decline in activity. The mean slope across the verbal tasks was +0.124, suggesting a mild overall increase. In the arithmetic task (AT) alone, six participants (A, B, D, E, F, and G) had positive slopes, and two (C and H) had negative slopes. The mean slope during the arithmetic task was +0.098, indicating increased cerebral blood flow during task execution in most participants. Across all tasks (WT1-AT), five participants (B, D, E, F, and G) showed positive slopes and three (A, C, and H) showed negative slopes. The overall mean slope was slightly positive (+0.107), suggesting a general trend toward increased prefrontal activation across the sequence of tasks.

## Discussion

Principal findings

In this study, we evaluated changes in oxygenated hemoglobin (Δoxy-Hb) in the prefrontal cortex (PFC) of mothers one month postpartum, who were clinically assessed as healthy, using near-infrared spectroscopy (NIRS). The mean Δoxy-Hb in the left prefrontal cortex (channels 13-22) showed an overall increasing trend from the verbal fluency tasks (WT1-WT3) to the arithmetic task (AT). However, three out of eight participants exhibited a gradual decline in activity with task repetition (Figure 2). In other words, while transient activation was observed at the beginning of the session, some mothers demonstrated a progressive reduction in prefrontal oxygenation during continuous task performance. This finding does not represent low baseline reactivity but rather a gradual decrease in responsiveness under sustained cognitive load-an observation that provides novel empirical evidence of neural fatigue.

Comparison with previous studies

This finding aligns with previous studies reporting altered neural activity during the postpartum period [16,17], yet it is unique in showing that even mothers evaluated as healthy can exhibit attenuation of prefrontal activation with sustained load. Earlier studies have indicated reduced PFC activity in postpartum women with depressive symptoms, anxiety, or childcare-related emotional stress [18,19]. In contrast, this study demonstrated that even in a “clinically healthy” group, the accumulation of fatigue and reduced energy resources can compromise the ability to maintain activation during task continuation. Therefore, prefrontal oxygenation responses may serve as an indicator of individual differences in recovery and endurance capacity in response to cognitive load, rather than merely reflecting group differences.

Clinical implications

Among the three participants who showed decreased activity (A, C, and H), participant C had a relatively high EPDS score (7 points), consistent with a mild depressive tendency and reduced neural activity. In contrast, participants A and H had very low EPDS scores (0 and 1, respectively) and were self-rated as healthy, yet they exhibited decreasing prefrontal activation with repeated and sustained tasks [8,9]. In participant C, the number of words generated fluctuated across verbal tasks, and the number of subtractions markedly declined in the subsequent arithmetic task, suggesting reduced cognitive resources due to task repetition. This pattern likely reflects a state in which neural fatigue has begun to manifest at the behavioral level [9,10]. Conversely, in participants A and H, task performance (word production and subtraction counts) was maintained despite declining neural activity, suggesting a state of “latent fatigue,” in which neural resources are being depleted without observable performance decline [5,18]. Thus, postpartum mothers may exhibit two distinct patterns of fatigue expression-overt and latent fatigue [1,16,17].

The finding that some participants maintained behavioral performance despite reduced neural activation indicates a dissociation between behavioral and neural levels of functioning [8,19]. Although external performance may appear preserved, neural fatigue may be progressing internally. This suggests the existence of “hidden fatigue vulnerability” among postpartum women [5,9]. The ability of NIRS to visualize such latent decreases in neural energy that are difficult to capture through self-report scales highlights its value as a physiological marker [13,18].

Clinically, this study does not aim to propose NIRS as a diagnostic tool. However, the finding that some mothers classified as healthy show reduced sustainability of prefrontal activation under cognitive load is noteworthy. The postpartum period is characterized by sleep deprivation and parenting-related stress, and self-administered scales such as the EPDS may not fully capture “subthreshold” fatigue or emotional vulnerability [20]. Therefore, monitoring PFC responses under cognitive load may offer a physiological means for early detection of latent fatigue and resource depletion. The pattern in which activation declines under load, even in apparently healthy individuals, may represent an early warning sign of future depressive symptom onset. Since depression can be prevented through early intervention [21], recognizing signs of fatigue and reduced resources even in mothers deemed healthy, and providing timely support, guidance, and environmental adjustments, is crucial for clinical practice.

Furthermore, declines in maternal mental health are known to be associated with an increased risk of child maltreatment [22,23]. The presence of mothers who show reduced sustainability of prefrontal activation under cognitive load suggests a potential risk for impaired emotional regulation under accumulated stress in daily life. Even when self-rated as healthy, early recognition of decreased neural activation in stressful contexts and providing support-such as counseling, rest, or parenting assistance-may contribute to the prevention of postpartum depression and, ultimately, to the primary prevention of child abuse.

Limitations and future directions

The present study has several limitations. First, the sample size was small (n = 8), limiting generalizability. Second, measurements were restricted to the left prefrontal cortex and did not capture whole-brain activity. Third, the statistical relationship between behavioral indicators (word and subtraction counts) and hemodynamic responses was not analyzed in detail. Future studies should incorporate graded task loads (varying difficulty and duration), multi-site measurements, and longitudinal assessments of the relationship between behavioral performance and cerebral hemodynamics. Integrating physiological indicators with measures such as household stress and sleep patterns will be essential to elucidate individual profiles of neural fatigue. Additionally, intervention studies are needed to verify whether early preventive support for mothers deemed healthy can help reduce the onset of depression, improve parenting outcomes, and lower the risk of child maltreatment.

## Conclusions

This study evaluated prefrontal brain activity in eight mothers one month postpartum who were clinically assessed as healthy, using near-infrared spectroscopy (NIRS). The results revealed that while transient activation was observed at the beginning of the tasks, approximately half of the participants showed a gradual decline in activation with task repetition and continuation. These findings suggest early signs of neural fatigue that cannot be detected through self-administered psychological scales.

The significance of this study lies not in recommending NIRS as a screening device but in visualizing the possibility that even mothers considered “healthy” may harbor latent fatigue and vulnerability in brain function. This finding underscores the importance of adopting a supportive approach that presumes the presence of “invisible fatigue” when working with postpartum mothers in clinical practice. Detecting early signs of fatigue before the onset of overt depression and providing timely interventions and environmental support may help protect the well-being of both mother and child and contribute to the primary prevention of child maltreatment.

## Figures and Tables

**Figure 3:**
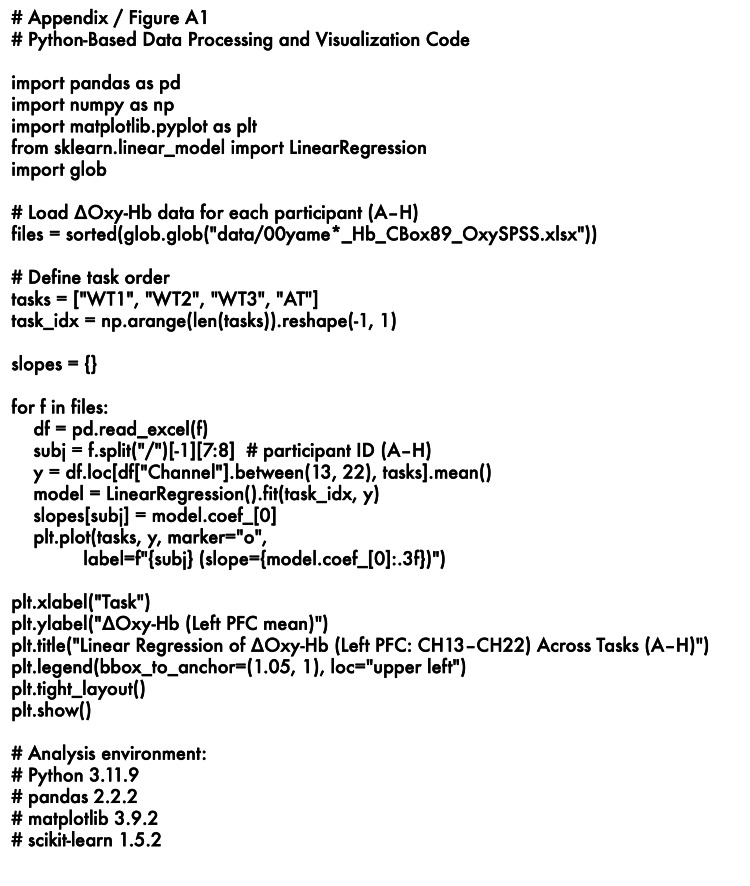
Python-Based Data Processing and Visualization Code Python script used for preprocessing and linear regression analysis of ΔOxy-Hb data (A–H). The code was executed using Python 3.11.9, pandas 2.2.2, matplotlib 3.9.2, and scikit-learn 1.5.2.
